# Observation of void formation patterns in SnAg films undergoing electromigration and simulation using random walk methods

**DOI:** 10.1038/s41598-021-88122-w

**Published:** 2021-04-21

**Authors:** Zhi Jin, Yu-An Shen, Yang Zuo, Y. C. Chan, S. H. Mannan, Hiroshi Nishikawa

**Affiliations:** 1 Graduate School of Engineering, Osaka University, Suita, Japan; 2 Joining and Welding Research Institute (JWRI), Osaka University, Ibaraki, 5600047 Japan; 3 Physics Department, School of Natural & Mathematical Sciences, King’s College London, Strand, London, WC2R2LS UK; 4 Department of Electronic Engineering, City University of Hong Kong, Tat Chee Avenue, Kowloon Tong, Hong Kong; 5 Present Address: Feng Chia University, Taichung, Taiwan (R.O.C.)

**Keywords:** Metals and alloys, Electrical and electronic engineering

## Abstract

With the ever-reducing sizes of electronic devices, the problem of electromigration (EM) has become relevant and requires attention. However, only the EM behavior of Sn–Ag solders within the solder joint structure has been focused on thus far. Therefore, in this study, a thin metallic film composed of Sn–3.5Ag (wt.%) was subjected to a current density of 7.77 × 10^4^ A/cm^2^ at a temperature of 15 °C to test the ability of existing EM models to predict the nucleation and evolution of voids generated by the resulting atomic migration. A computer simulation was then used to compute the coupled current distribution, thermal distribution, and atomic migration problems. It relied on an original random walk (RW) method, not previously applied to this problem, that is particularly well suited for modelling domains that undergo changes owing to the formation of voids. A comparison of the experimental results and computer simulations proves that the RW method can be applied successfully to this class of problems, but it also shows that imperfections in the film can lead to deviations from predicted patterns.

## Introduction

Continually evolving requirements for high speeds, increased functionality and lower weight are forcing circuits to become smaller. This results in high current density within solder joints. According to the 2003 International Technology Roadmap for Semiconductors, a significant downsizing in the flip chip (FC) package size is anticipated. It is also expected that the bump size, pad size, and pitch will be reduced. In 2007, the diameter of FC micro-bumps was as small as 20 μm. In the microelectronics industry, each solder joint was originally designed to carry 0.2 A, and this value will double in the near future^1^. This means that the average current density may approach 10^4^ A/cm^2^ in a solder joint with a diameter of 50 µm^2^. Joule heating, which increases as the square of current density, is regarded as one of the most important reliability factors because the temperatures in the solder joint rise to 100 °C, which is close to 76% of the melting point of a eutectic Sn–Ag–Cu solder^3^. Electromigration (EM) will therefore seriously degrade the reliability of electronic packaging when high current density and temperature accelerate atomic diffusion in the solder joints^4,5^.

Yeh et al. reported that voids nucleated near the regions where the current was applied and then extended across the entire eutectic Sn–Pb solder joints under a current density of 2.25 × 10^4^ A/cm^2^ at 150 °C^6^. This is also observed in Sn–4.0Ag–0.5Cu solder bumps at a current density of 3.67 × 10^3^ A/cm^2^ at 146°C^7^. However, the presence of a daisy-chained series of FC solder joints connected by a Cu or Al wire significantly complicates the EM analyses further because the effects of the solders and the wires both have to be considered.

Most studies have focused on the EM behavior of Sn–Ag solders only within the structure of a solder joint. However, Belch and Herring first studied EM in a material with an edge drift structure, that is, with a thin film structure, which allows for the fundamental mechanisms underpinning EM to be observed while eliminating the complications caused by 3-D structures^8^. The setup used in this study also employs a thin film while simultaneously eliminating the effects due to heterogeneous junction interfaces. Zhu et al. previously carried out EM tests in a Sn–3.5Ag thin film with the same thin-film structure for the observation of void growth at the cathode^9^; it was found that after EM, the growth path differed from that predicted by the finite element analysis (FEA) simulations. This work improves on the work done by Zhu et al. by using an improved simulation tool based on the random walk (RW) approach, and by studying the void pattern evolution over an extended time period rather than only at the end of the experiment.

## Experiment results

**Figure 1:**
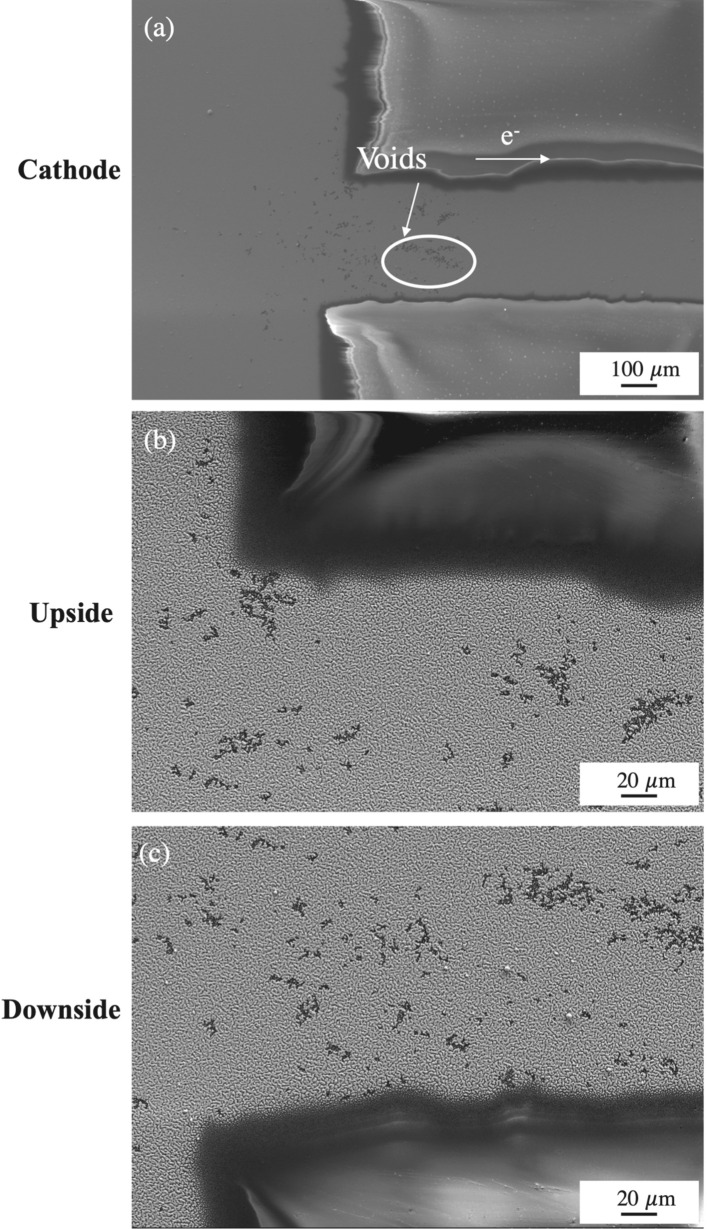
Morphology of the cathode after uninterrupted EM for 650 h (**a**) Overall morphology (**b**) Upside region of the cathode (**c**) Downside region of the cathode.

**Figure 2:**
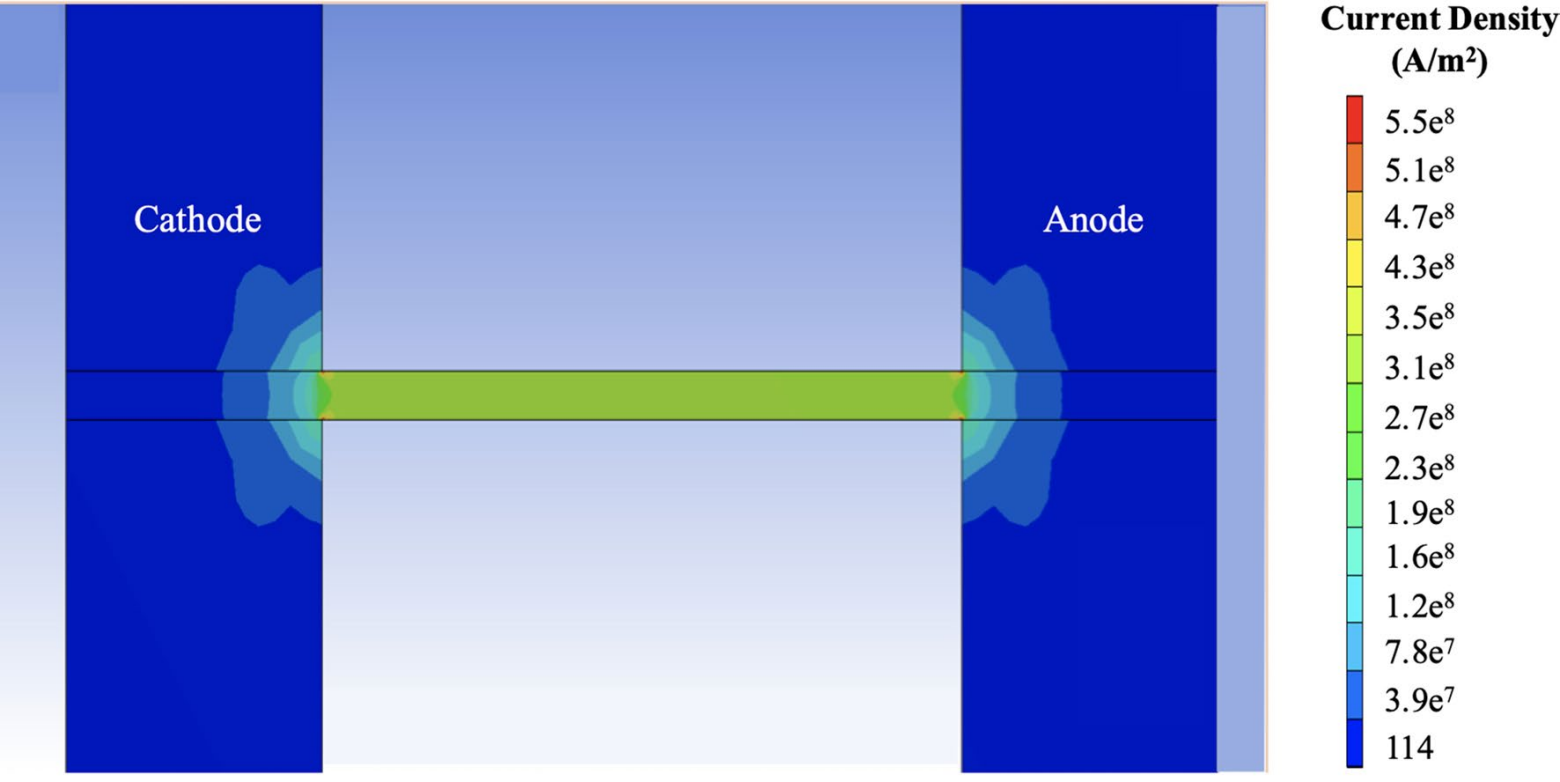
Current density distribution of the sample.

The microstructure evolution of cathode side of the sample after uninterrupted EM for 650 h are shown in Fig. 1. Several void clusters were observed (Fig. 1a). Some clusters exhibited a diagonal alignment similar to that observed by Zhu et al.^9^ whereas others appeared to be randomly dispersed. To better understand the factors influencing void distribution, the cathode was divided into upside and downside zones. The void density was larger on the downside than on the upside, as shown in Fig. 1b,c. Because of the driving force for EM, atoms on the cathode moved toward the anode, leaving vacancies behind. The electron wind force is described below^10^:

1 $$ \vec{F}_{em} = Z^{*} e\vec{E} $$

where *e* is the electron charge, $\vec{F}_{em}$ is the driving force for EM, $Z^{*}$ is the effective charge or effective valence, and $\vec{E}$ is the electric field. This equation shows how the driving force is directly related to $\vec{E}$, which in turn is proportional to the current density under direct current conditions. Figure 2 shows the magnitude of the current density across the strip. Note that current crowding occurs at a corner, with the current density reaching levels approximately twice the average current density in the strip. Therefore, the driving force for EM in Sn atoms in the current crowding areas is much higher, and the voids generated in these locations should appear first in the absence of other factors.

**Figure 3:**
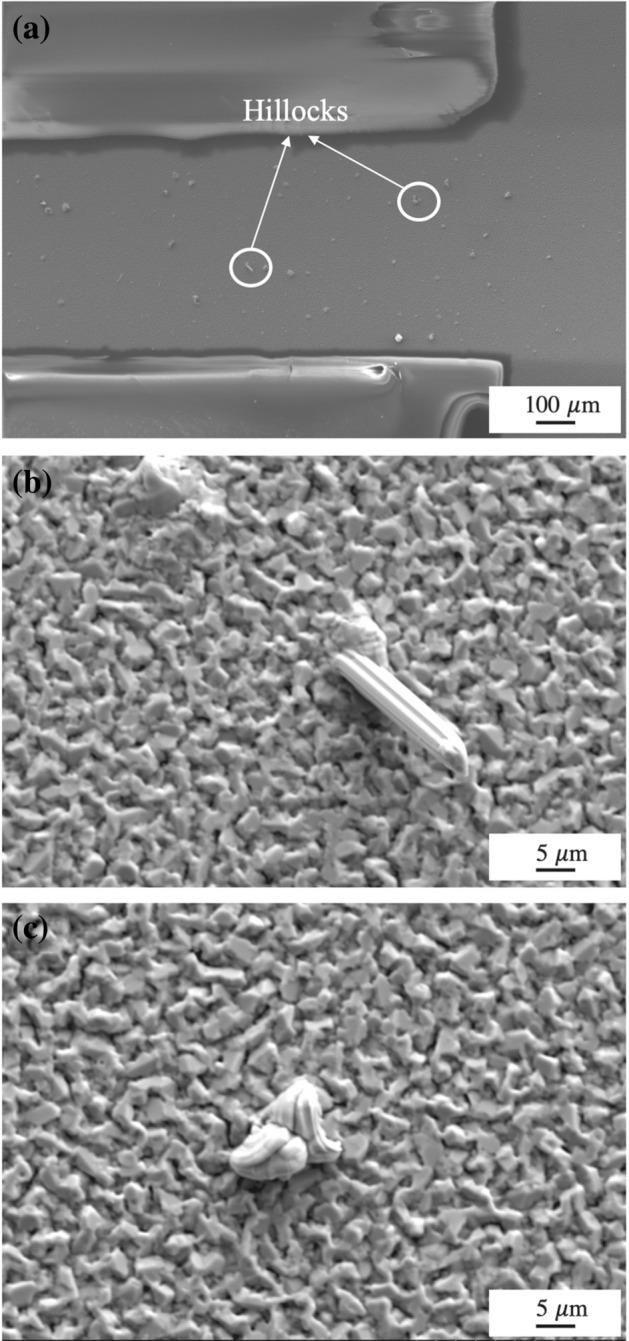
Morphology of the anode after uninterrupted EM for 650 h (**a**) Overall morphology, (**b**) and (**c**) Hillocks generated in the anode.

Figure 3a shows the morphology of the anode. Hillocks were observed on the surface of the thin film, as shown in Fig. 3b,c, respectively. The generation of Sn hillocks is considered a surface relief phenomenon caused by stress generation and relaxation. There are two essential conditions for Sn-hillock growth. First, a continuous compressive stress must be generated to maintain the driving force for EM. Second, stress must be relaxed through the growth of hillocks^11^. In the EM test, the electron wind force carries Sn atoms from the cathode to the anode, creating a compressive-stress field on the anode side. Once the stress reaches a critical level, the Sn atomic flow breaks through localized weak spots on the oxide surface at the anode side to form Sn hillocks^12^.

**Figure 4:**
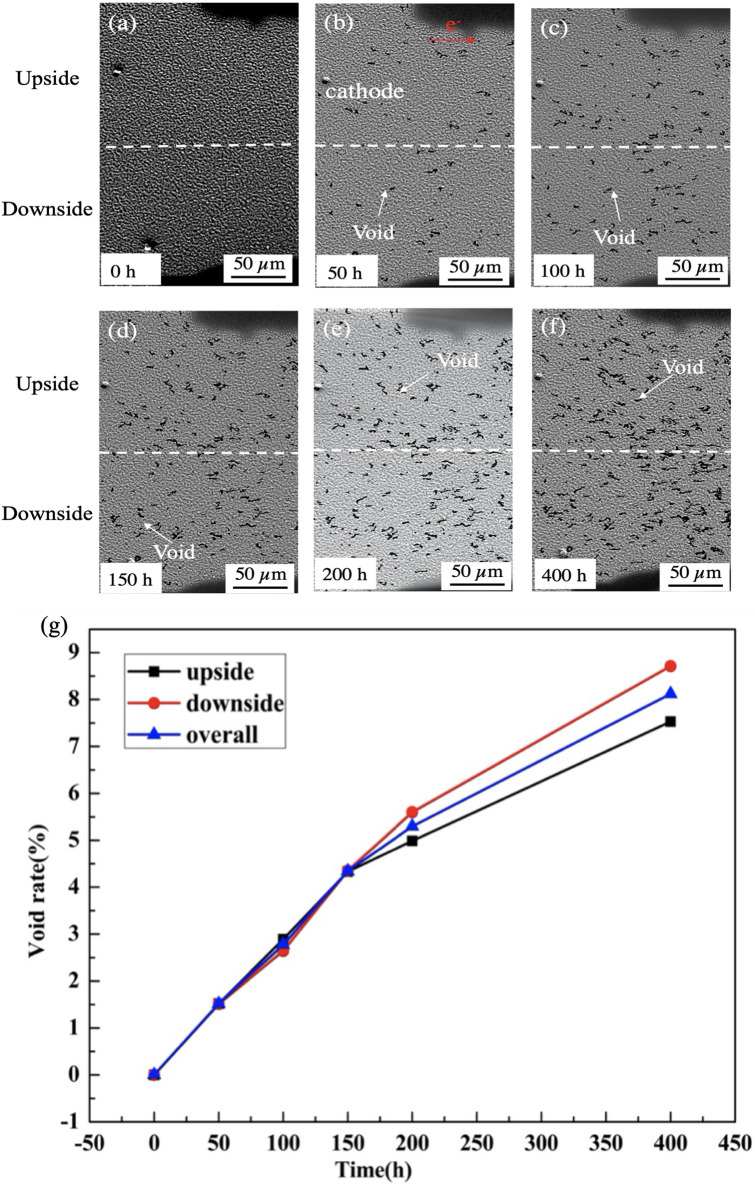
The morphology of the Sn–3.5Ag strip at different times (**a**) Initial sample (**b**) EM for 50 h (**c**) EM for 100 h (**d**) EM for 150 h (**e**) EM for 200 h (**f**) EM for 400 h (**g**) the rate of void growth on the cathode.

In the study by Zhu et al.,^9^ only the overall void distribution was observed, and not the order of void nucleation or the void-growth pattern after nucleation. Regular observations of void evolution were therefore made during this experiment. Figure 4 shows images of the cathode of the second sample after different periods of EM between 0 and 400 h. Figure 4a shows the initial structure, homogeneously deposited, and without any voids on the glass. After EM for 50 h, a small number of voids could be observed as in Fig. 4b, and this number progressively increased with time. Figure 4g presents the void fraction, calculated by dividing the void area by the entire area of the strip. This indicates that the growth rate of voids for the entire strip was the highest during the early stages of the experiment. Moreover, the growth rates of the upside and downside zones were observed to be the same during the first 150 h, as would be expected from the overall symmetry of the cathode area. The voids appeared homogeneously on the strip during this period. However, the void density in the downside zone was higher than that in the upside zone after 200 h, and the disparity increased thereafter.

**Figure 5:**
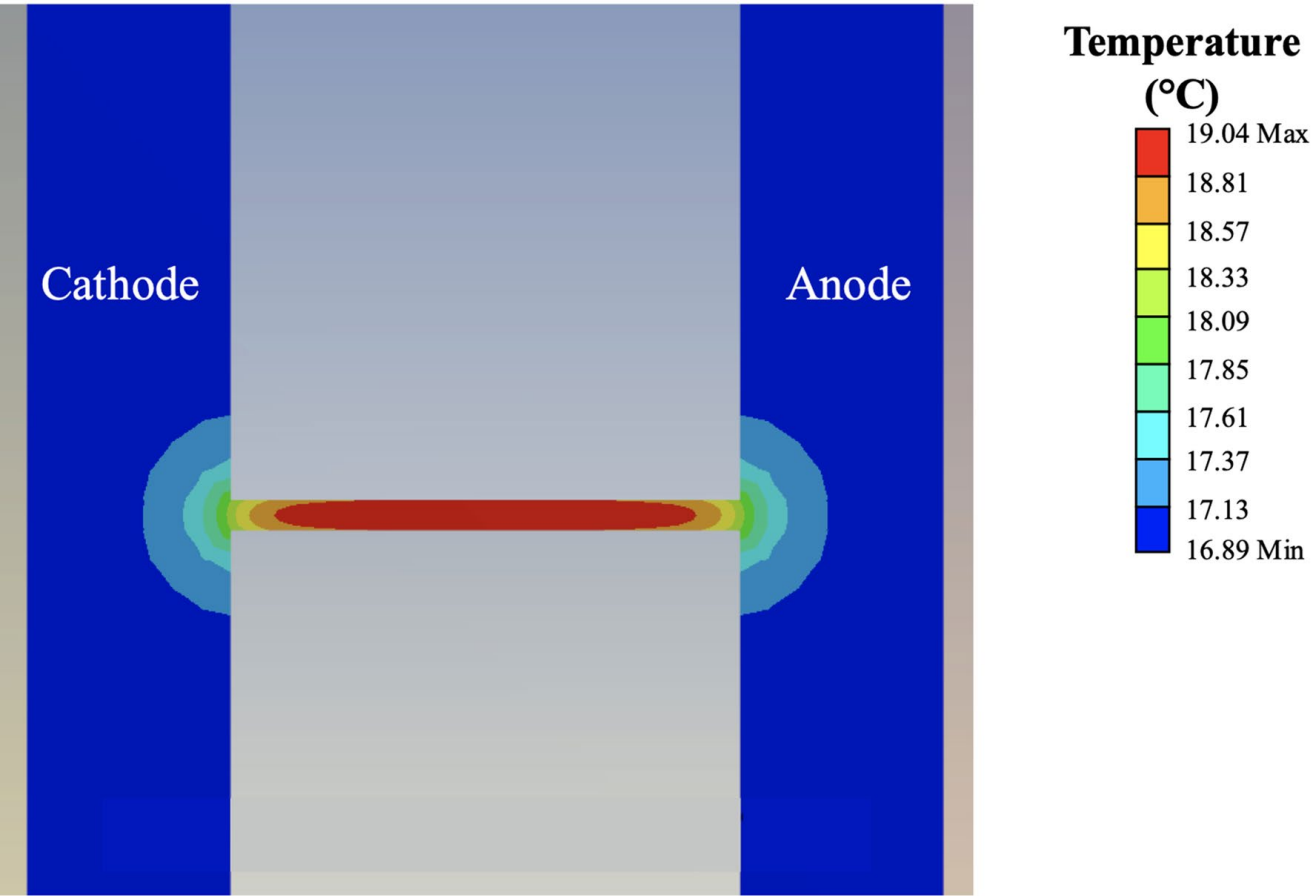
Temperature distribution of the sample.

If void nucleation is considered to be affected only by the magnitude of $\vec{F}_{em}$ and therefore the instantaneous current density, then voids should grow diagonally from the upside zone to the downside zone on the edge of the strip. There should be no other disparity between the upside and downside zones according to the previous simulation predictions^9^. However, the experimental results shown in Fig. 4 do not agree with this conclusion. Therefore, other factors need to be considered in addition to the instantaneous current density. It is well known that the grain orientation affects the diffusion speed of Sn atoms during the EM test^13^. To avoid the effect of the preferred grain orientation on Sn diffusion, electron-beam physical vapor deposition was applied. During deposition, individual grains are oriented randomly, unlike in traditional solder joints, and the Sn grain orientation before the test is shown in Fig. S1. Therefore, the effect of the preferred grain orientation on void generation can be ignored. The distribution of Ag is shown in Fig. S2. It was found that Ag_3_Sn intermetallic compounds (IMCs) are homogeneously distributed along the strip, comprising crystals smaller than those in traditional solder joints. Therefore, the stress gradient caused by the IMCs can also be ignored. Temperature is another important factor that affects the speed of atomic migration. Figure 5 shows the temperature distribution of the overall sample. The maximum temperature rise was 4.04 °C where was on the middle of stripe since the simulation boundary condition was set as 15 °C. And the minimum temperature was found on the end of the stripe; therefore, a temperature difference of 2.15 °C was generated depended on the simulation result. Since the stripe is a symmetrical structure, the length from end of stripe to the middle part was 2.5 mm. Thus, the temperature gradient was calculated as 8.6 °C/cm, two orders of magnitude below that required to trigger the thermomigration effect^14^. Note that temperature indirectly contributes to void nucleation because of the dependence of atomic diffusion on temperature. However, the previous analysis neglected the role of temperature and focused only on the driving force for EM.

The analysis by Zhu et al.^9^ showed that the divergence of the atomic flux, Div($\vec{J}_{{{\text{EM}}}}$) (i.e., the net rate at which atoms leave a given region) is given by

2 $$ Div(\vec{J}_{{{\text{EM}}}} ) = \left( {\frac{{E_{a} }}{{KT^{2} }} - \frac{1}{T}} \right)\frac{{C_{a} Z^{*} e\rho }}{KT}D_{0} \exp \left( { - \frac{{E_{a} }}{KT}} \right) \vec{J}_{e} \cdot \nabla T, $$

where *E*_*a*_ is the activation energy, *C*_*a*_ is the atomic concentration, $Z^{*}$ is the effective charge number, *e* is the electron charge, $\rho$ is the resistivity, *D*_*0*_ is the pre-exponential factor, *K* is the Boltzmann constant, *T* is the average thermal energy, and $\vec{J}_{{\text{e}}}$ is the current density applied in the experiments. This can be further simplified under the conditions of an approximately uniform temperature to

3 $$ {\text{Div}}(\vec{J}_{{{\text{EM}}}} ) \propto \vec{J}_{e} \cdot \nabla T, $$

The results shown in Fig. 4 were expected to indicate that voids nucleate first at the corners where the magnitude of $\vec{J}_{{\text{e}}}$ is the highest^9^. However, this was not observed; instead, voids appeared randomly across the cathode area. A new simulation method is required to predict void generation at the cathode, which takes into account $\nabla T$ as well as $\vec{J}_{{\text{e}}}$.

The evolution and growth of voids with time are also required to be investigated in detail. It is unclear whether the voids gradually grow from the initial nucleation site, or whether new voids are generated near the initial void, merging to form larger voids. Figure S3 indicates that voids, once nucleated, do not move or change shape. Instead, neighboring grains form preferential sites for further void nucleation and eventually merge. Thus, sub-grain resolution is eventually required in the simulations. The FEA method is limited, as higher mesh resolution requires higher memory requirements and causes convergence problems, and so a new approach is required that can be adapted for grain and sub-grain resolutions.

## Simulation results

Several simulations have analyzed void evolution assuming that the highest current density is located at the site of the first void^15–17^. Equation (3), however, shows that the current density and the temperature distribution are both required to determine the site of the maximum depletion of atoms and hence the first void. The voltage distribution across domain $V$, the electric field $\vec{E}$, and the current distribution $\vec{J}_{{\text{e}}}$ is computed using Laplace’s equation as follows:

4 $$ \nabla^{{2}} V = 0, $$

5 $$ \vec{E} = - \nabla V, $$

6 $$ \vec{J}_{e} = \sigma \vec{E}, $$

where ${\upsigma }$ is the electrical conductivity of the metal. Once $\vec{J}_{{\text{e}}}$ has been calculated, the temperature *T* can be determined from the heat equation assuming that the temperature across the domain rapidly reaches a steady state relative to the time scales of the experiment using the following equation:

7 $$ \nabla^{2} T = \frac{1}{k\sigma }\left| {\vec{J}_{e} { }} \right|^{2} , $$

where *k* is the thermal conductivity of the metal. Calculations show that heat conduction through the metal dominates heat flow through convection or through the glass substrate^9^.

**Figure 6:**
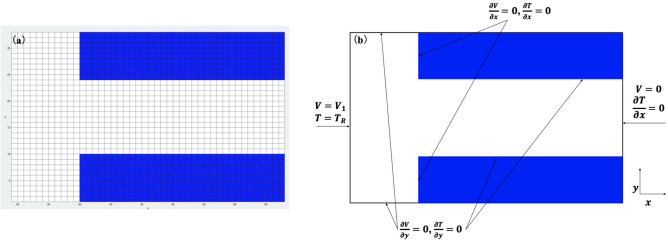
Model of simulation (**a**) cells of the strip (**b**) boundary conditions for the simulation.

The boundary conditions for the simulation are shown in Fig. 6a,b. The white cells indicate the shape of the Sn–3.5Ag strip, and the blue-colored cells at the top and bottom represent the glass edges, as shown in Fig. 6a. The boundary conditions (Fig. 6b) for *V* are $V = V_{1}$ on the left-hand side (l.h.s.) of the domain, $V = 0$ on the right-hand side (r.h.s.) of the domain, and $\partial V = 0$ on the other boundaries.

8 $$ \frac{\partial V}{{\partial x}} = 0, \frac{\partial V}{{\partial y}} = 0 $$

The above equations represent the voltage gradients at the vertical and horizontal boundaries, respectively, reflecting the fact that there is no current flow in or out of the domain apart from the l.h.s. and r.h.s. boundaries. We assume that all boundaries, including void boundaries, are either horizontal or vertical. Further, the boundary conditions are $T = T_{R}$ (room temperature, set at 15 °C) at the l.h.s. and r.h.s. boundaries and $\partial T = 0$ at the other boundaries.

The FEA simulation data for the domain (Fig. 5) indicate a maximum temperature difference of 4.04 °C. The parameters used for the calculations are listed in Table S1. These results confirm that temperature is relatively uniform across the strip and therefore, the simplifying assumptions leading to Eq. (3) can be used to calculate the divergence of atomic flux^9^.

To calculate the atomic flux divergence, the domain was split into square cells, as shown in Fig. 6a. These cells were artificially constructed and used to track local variables and did not represent individual grains. The use of parallel processing techniques in the future should allow sub-grain resolution of the domain. Each cell was given a label, *i*, and $\vec{J}_{{\text{e}}}$ and $\nabla T$ were calculated at each site to give the flux divergence $F_{ij}$ at site $i$ in step $j$ as:

9 $$ F_{ij} = \left( {\vec{J}{\text{e}} \cdot \nabla T} \right)_{ij} , $$

Here, $F_{ij}$ is proportional to the rate at which atoms leave the site; hence the number of atoms that leave a site in time period $\Delta t$ is given by:

10 $$ \Delta N = aF_{ij} \Delta t, $$

where *a* is the geometrical constant in this case, which is the same for each equally sized cell in the domain and can hence be set to 1.

The algorithm used to predict the void nucleation order in this study proved more beneficial compared to previous attempts because of two reasons: Eq. (9) considered the thermal effects, and Eq. (10) was integrated numerically to include the total number of atoms that left each site, not only the site of instantaneous maximal flux divergence. When the number of atoms that had left a site reached the critical threshold, $N_{c}$, the cell was removed from the domain and transformed into a void. The algorithm was constructed such that the value of $N_{c}$ was not required to predict the order of void nucleation. If $N_{c}$ is known, the time of the appearance of the first void can be determined. The algorithm proceeded as follows:

*Step 1*: The 1st void nucleation site was calculated by finding the maximum value of $F_{i1}$ in the initial geometry without voids. This maximum value was denoted by $F_{m1}$.

11 $$ F_{m1} = \mathop {\max }\limits_{i} F_{i1} , $$

where index *i* was used for all cells in the domain. The first void nucleated after time $t_{\left( 1 \right)}$:

12 $$ N_{c} = F_{m1} t_{\left( 1 \right)} $$

*Step 2*: The location of the subsequent void was determined. First, the flux divergences were recalculated for each cell, with the initial void now present. The void to nucleate subsequently would be the first cell to reach $N_{c}$, taking into account both the atoms that left the cell in the first step, and the number of atoms that left the cell in the second step. At each cell, the time required for $t_{i}$ was calculated as follows:

13 $$ N_{c} = F_{i1} t_{\left( 1 \right)} + F_{i2} t_{i} \Rightarrow t_{i} = \frac{{N_{c} - F_{i1} t_{\left( 1 \right)} }}{{F_{i2} }} \Rightarrow \frac{{t_{i} }}{{t_{\left( 1 \right)} }} = \frac{{F_{m1} - F_{i1} }}{{F_{i2} }} $$

The next cell to nucleate a void was given by the smallest $t_{i}$, denoted by $t_{\left( 2 \right)}$:

14 $$ \frac{{t_{\left( 2 \right)} }}{{t_{\left( 1 \right)} }} = \mathop {\min }\limits_{i} \frac{{F_{m1} - F_{i1} }}{{F_{i2} }}. $$

*Step 3*: Step 2 was repeated, with the flux divergences recalculated each time with the geometry updated with the location of all previously identified voids. Therefore, Eq. (13) changed as follows:

15 $$ \frac{{t_{\left( n \right)} }}{{t_{\left( 1 \right)} }} = \mathop {\min }\limits_{i} \frac{{F_{m1} - \mathop \sum \nolimits_{j = 1}^{n - 1} F_{ij} t_{\left( j \right)} /t_{\left( 1 \right)} }}{{F_{in} }} $$

Note that such use of the ratio of time steps $t_{\left( 1 \right)}$ (and hence $N_{c}$) was not required before to be explicitly computed.

The void nucleation pattern could be determined by repeatedly calculating the $F_{ij}$ values and updating the geometry. The FEA method is suitable for such calculations in simple domains, but better results can be obtained by decreasing the cell size. As the complexity of the domain increases, FEA methods become limited by the amount of memory required to carry out calculations. RW-based methods for solving partial differential equations (PDEs), such as Eq. (4) and Eq. (7), have several advantages in this type of simulation^18^. These include negligible memory requirements, ease of implementation on parallel computer architectures (e.g. cloud based computing or multiple GPU nodes), and the ability to compute the solution locally without needing to solve the PDE over the whole domain. The latter advantage requires the use of stochastic calculus, but for proof of concept, a simple and intuitive RW method is used (implemented in MATLAB), which relies on the approach outlined by Chandrasekhar^19^.

This approach relies on the formal similarity between Fick’s laws of diffusion at steady state for the concentration of a swarm of non-interactive particles, and the PDEs in Eq. (4) and Eq. (7). Recall that at steady state, the concentration of particles undergoing diffusion, $C$, is calculated by the following:

16 $$ \nabla^{2} C = S, $$

where *S* is a source term for particles, which is set to 0 for Eq. (4) and set as $\frac{1}{k\sigma }\left| {\vec{J}_{e} { }} \right|^{2}$ for Eq. (7). A constant boundary condition is achieved through the release of a constant number of particles into the domain from a boundary. The zero boundary condition is achieved through the absorption of particles as they encounter the boundary. Finally, $\partial V = 0$ and $\partial T = 0$ boundary conditions are achieved through the reflection of particles from the boundaries. Hence, Eq. (4) is solved by releasing particles at the l.h.s., performing RW simulations while they are inside the domain and absorbing them when they reach the r.h.s. of the domain. The number of particle visits to each cell is recorded and is found to be proportional to *C*. Equation (7) is solved by releasing particles from each cell, and the number of particles released is proportional to the computed value of $\left| {\vec{J}_{e} { }} \right|^{2}$, which is computed from the solution to Eq. (4). The solutions for $\vec{J}_{e}$ and $\nabla T$ are used to find $F_{ij}$ values from Eq. (9) and steps 1–3 are followed to determine the order of void nucleation.

**Figure 7:**
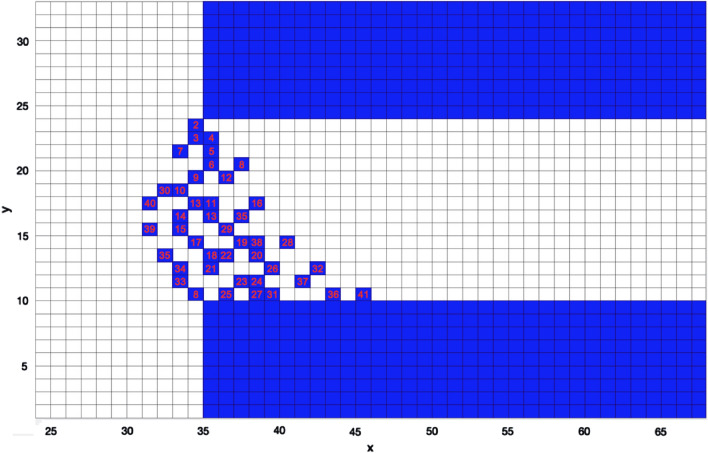
Random walk simulation results of void distribution on the strip.

**Figure 8:**
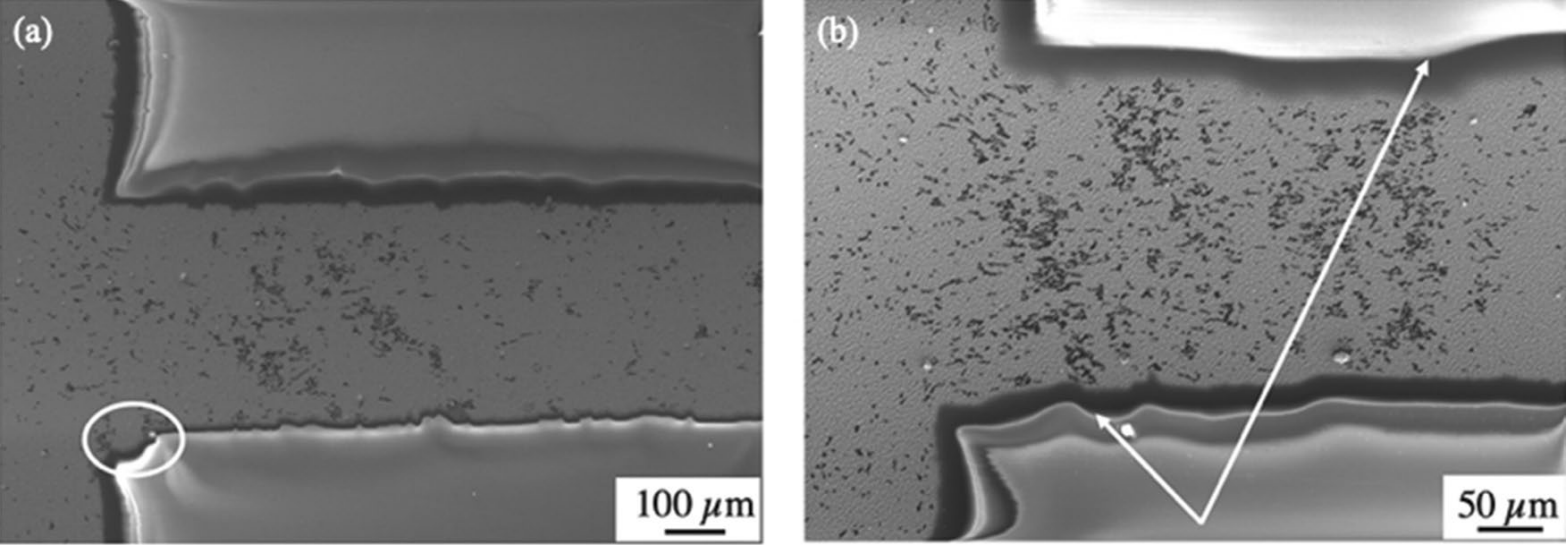
Imperfections in the film (**a**) shape of the sample after uninterrupted EM for 650 h (**b**) shape of the sample checked at regular intervals.

Using the simulation method described above, Fig. 7 shows the void nucleation pattern expected on the cathode. The strip was composed of white cells. The number of labeled cells on the strip represents the order of voids generated in the simulation and can be compared to the distribution of voids formed in the physical EM experiment. It was found that the asymmetry between the density of voids generated on the downside and upside was naturally generated. In addition, the simulation results indicated that the voids formed irregular diagonal patterns, similar to those found in the experiment. Therefore, the new simulation captured the essential features of the experimentally observed void pattern. However, the diagonal patterns in Fig. 8a emerging at the corner are not as pronounced as those in the interior of the strip, which was possibly because of imperfections in the film. As shown in Fig. 8b, some diagonal clusters formed at kinks in the film edges, while the cluster on the r.h.s. of the image appears to be associated with a significant widening of the strip. All of these defects in the experimental sample may cause variations in the simulation results.

## Conclusion

Thin film test specimens composed of Cr/Sn–3.5Ag on a glass substrate were used to observe the void growth and evolution on the cathode side of the strip. This structure was simplified when compared with the flip-chip and allowed the underlying void formation equations to be tested. The growth of the voids was recorded via scanning electron microscopy (SEM). The experimental results were compared with the simulation results.

The growth rate of voids over the entire strip was the highest in the early stages, and the growth rates in the upside and downside regions were identical during the first 150 h. A disparity between the upper and lower sides was identified after 150 h. Additionally, the experiments showed that once a void was formed, it remained in place and further growth occurred as neighboring grains transformed into voids. These voids finally merged to form larger voids.

By integrating the flux of the atoms leaving their sites, a more realistic pattern of void formation was found, compared to algorithms that rely on only computing the maximal current density. The “Random Walk” (RW) method was demonstrated to be a viable method of solving the PDEs relevant for EM phenomena, so that future work can use cells with dimensions similar to those of the grains being simulated.

## Methods

**Figure 9:**
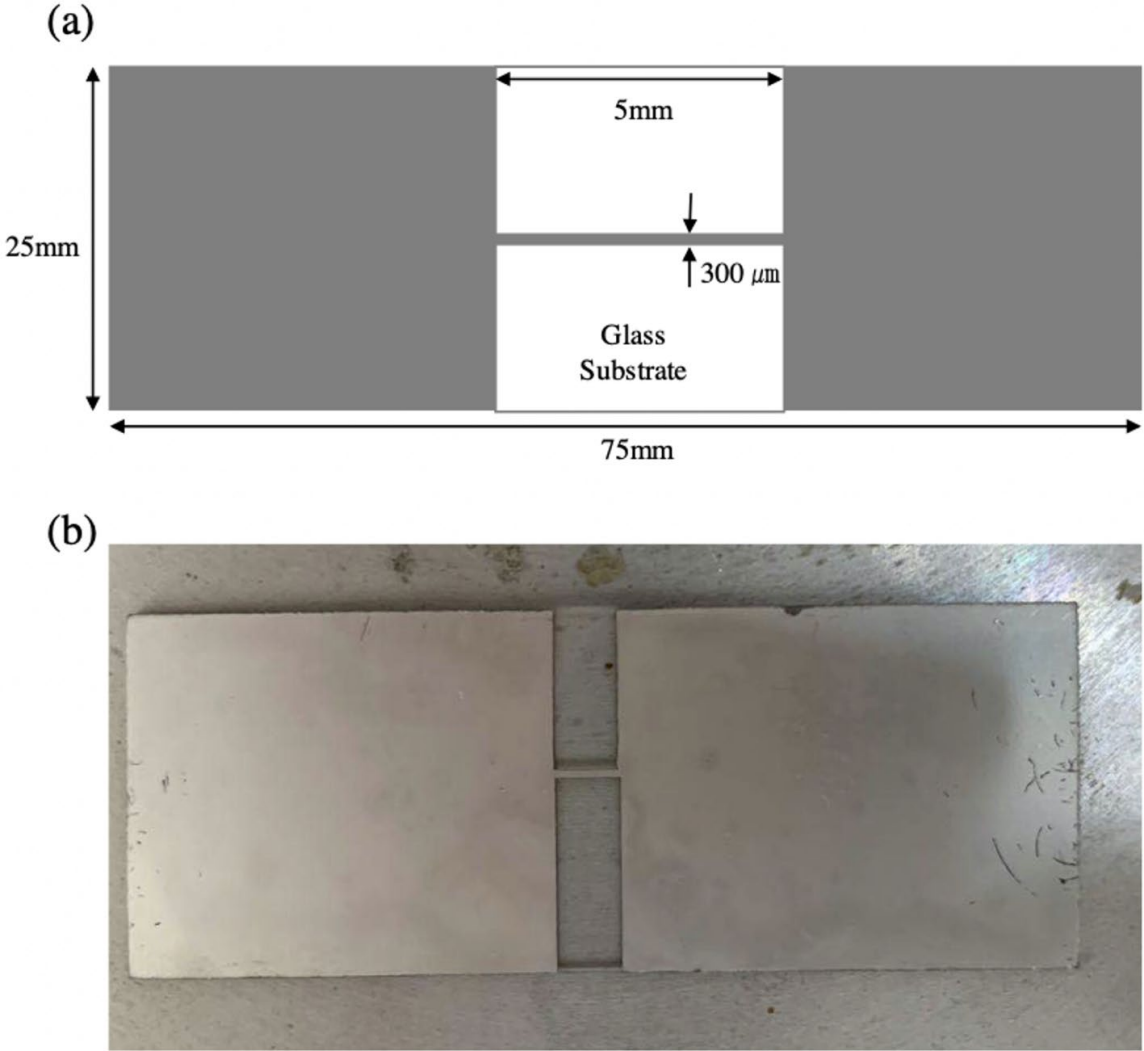
Schematic of the sample (**a**) plan view of the substrate after deposition, (**b**) experimental sample.

A standard microscope glass slide with dimensions of 75 × 25 × 1 mm served as the substrate. It was cleaned using solvent wipes, followed by rinsing with acetone and deionized water. Kapton tape was used as a pattern mask to define the specific geometry of the thin films during evaporation deposition (UEP-2000 OT-H/C, ULVAC, Japan). The deposition process was conducted at a rate of 0.1 nm/s in a vacuum chamber with a pressure of 10^−6^ mbar. The line width was set at 300 µm to avoid shadow effects. The line length was 5 mm, which was sufficiently long to eliminate the Blech effect associated with short strips. A plan view of the sample is presented in Fig. 9a. A Cr film with a thickness of 5 nm was coated as an adhesion layer on the glass substrate for the following layer of Sn–3.5Ag alloy, with a thickness of 300 nm. The samples used in the experiment are shown in Fig. 9b.

For the EM test, two clips connected to the power source were used to clamp the sample on the left and right sides, ensuring uniform current flow near the narrow strip. A copper plate was set below the EM-tested sample to drain the heat produced by Joule heating and limit the temperature rise in the joint. Two samples were prepared for the observation of void growth at the cathode during EM at a current density of 7.77 × 10^4^ A/cm^2^, and the experiment was conducted at a temperature of 15 °C. One sample was checked at regular intervals, whilst the other was subjected to uninterrupted EM for 650 h. The microstructural changes of the thin films were observed via field effect scanning electron microscopy (FESEM, Hitachi SU-70, Japan).

The RW simulations for void nucleation and growth were coded using MATLAB. Additionally, the electrical and thermal distributions in the EM-tested sample were independently evaluated using FEA (Ansys Workbench 13). The geometry of the sample is illustrated in Fig. S4. The parameters and boundary conditions are listed in Table 1.

**Table 1:** Parameters for the FEA simulation.

Given parameters	Values
Strip dimension	*l*: 5 mm/*w*: 300 μm/*h*: 300 nm
Glass dimension	*l*: 75 mm/*w*: 25 mm/*h*: 1 mm
Copper dimension	*l*: 125 mm/*w*: 40 mm/*h*: 5 mm
Applied voltage	1.3 V
Current	69.9 mA
Solder thermal conductivity	630 W/(m K)
Glass thermal conductivity	0.96 W/(m K)
Solder and glass emissivity	0.94
Copper emissivity	1
Gap between the copper block and the glass substrate	0.1 μm

## Supplementary Information

Supplementary Information.

## Data Availability

All data generated or analyzed during this study are included in this published article (and its Supplementary Information files).
