# Supplementary material for: Observation of void formation patterns in SnAg films undergoing electromigration and simulation using random walk methods
Source: Sci Rep. 2021 Apr 21;11:8668. doi: 10.1038/s41598-021-88122-w (PMC8060422; doi:10.1038/s41598-021-88122-w)
Supplement: Supplementary file 1 — Supplementary Information. [file 41598_2021_88122_MOESM1_ESM.pdf]

Observation of Void Formation Patterns in SnAg Films undergoing Electromigration and Simulation using Random Walk Methods

Zhi Jin[1, 2, 3*], Yu-An Shen[2#], Yang Zuo[3], Y.C. Chan[4], S.H. Mannan[3*], Hiroshi Nishikawa[2*]

1. Graduate School of Engineering, Osaka University, Suita, Japan

2. Joining and Welding Research Institute (JWRI), Osaka University, Ibaraki, 5600047, Japan

3. Physics Department, School of Natural & Mathematical Sciences, King's College London, Strand, London WC2R2LS, UK

4. Department of Electronic Engineering, City University of Hong Kong, Tat Chee Avenue, Kowloon Tong, Hong Kong

* Corresponding Author: Zhi Jin, S.H. Mannan, Hiroshi Nishikawa

This author is currently an Assistant Professor at Feng Chia University, Taichung Taiwan (R.O.C)

E-mail: jinzhi711@gmail.com, samjid.mannan@kcl.ac.uk, nisikawa@jwri.osaka-u.ac.jp

Supplementary Information

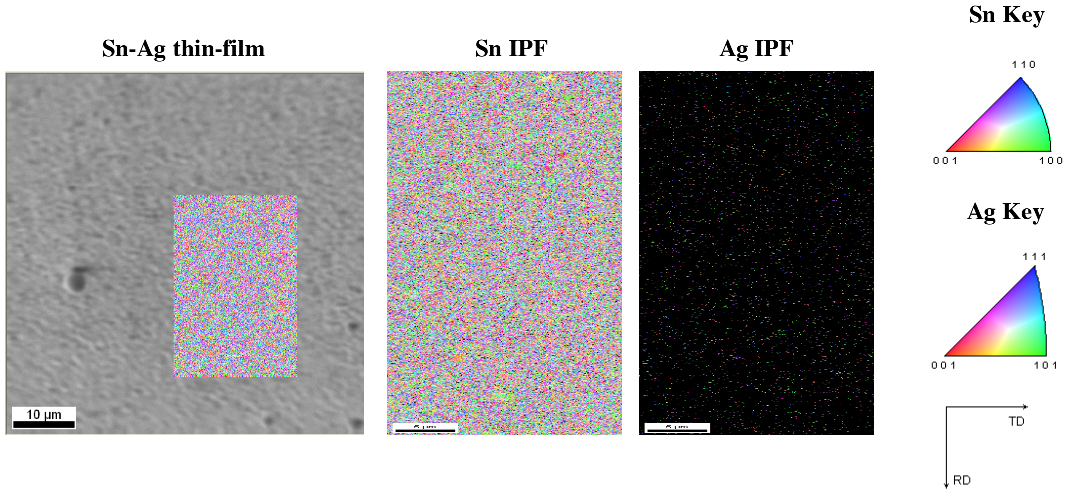

Figure S1. Grain orientation of Sn and Ag in the Sn-3.5Ag strip before the EM test

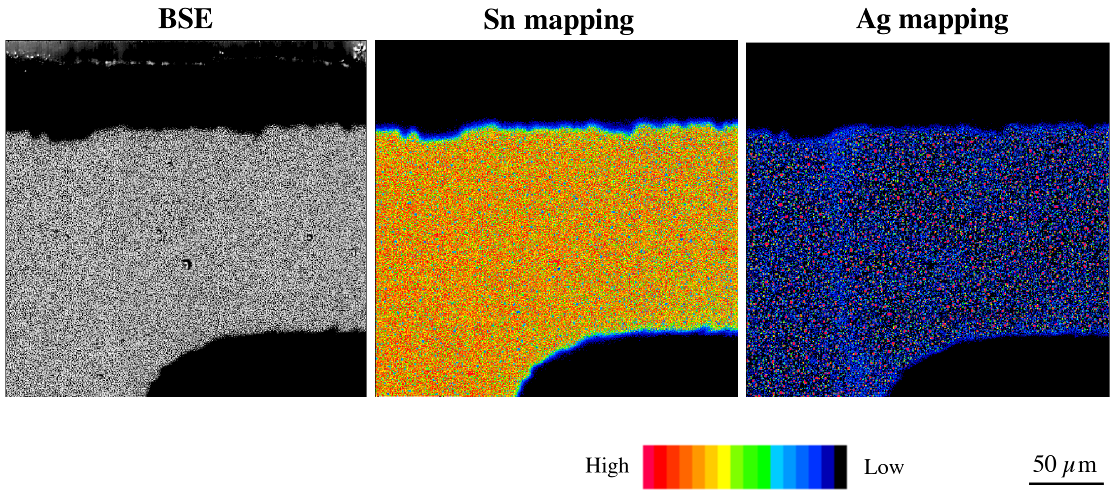

Figure S2. Distribution of Ag on the strip before the EM test

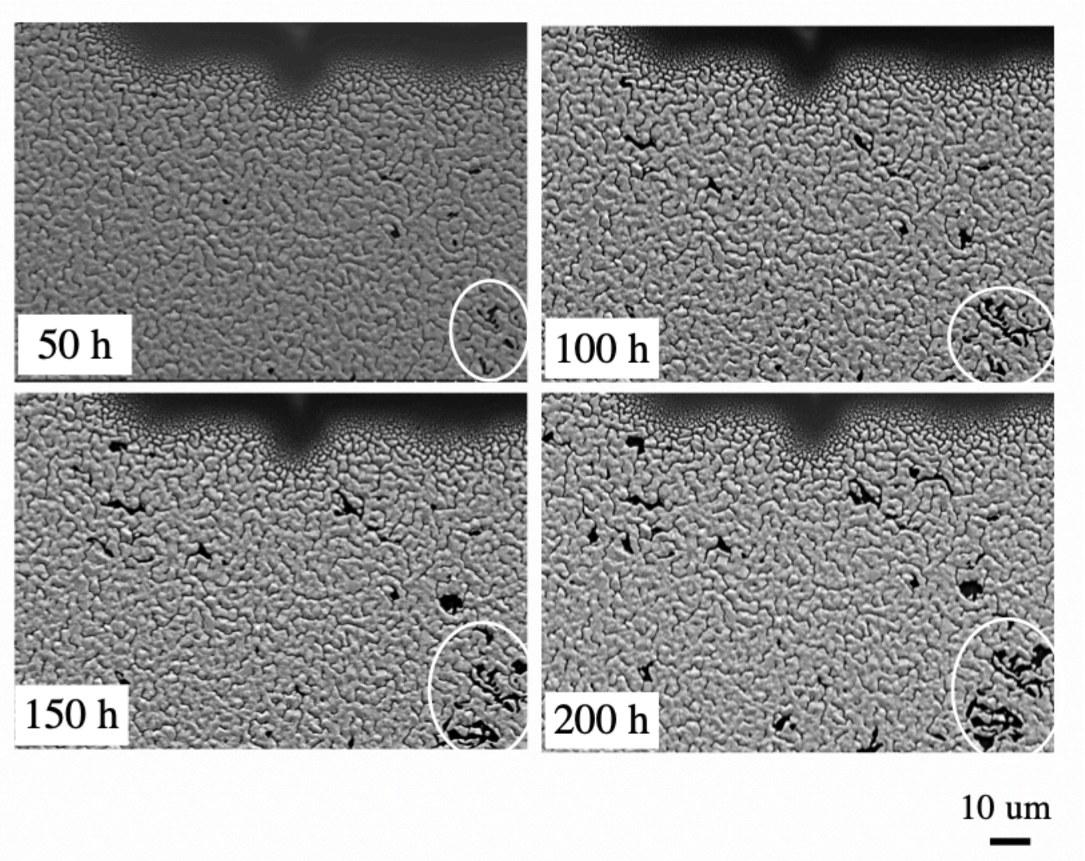

Figure S3. Evolution of voids during the EM test

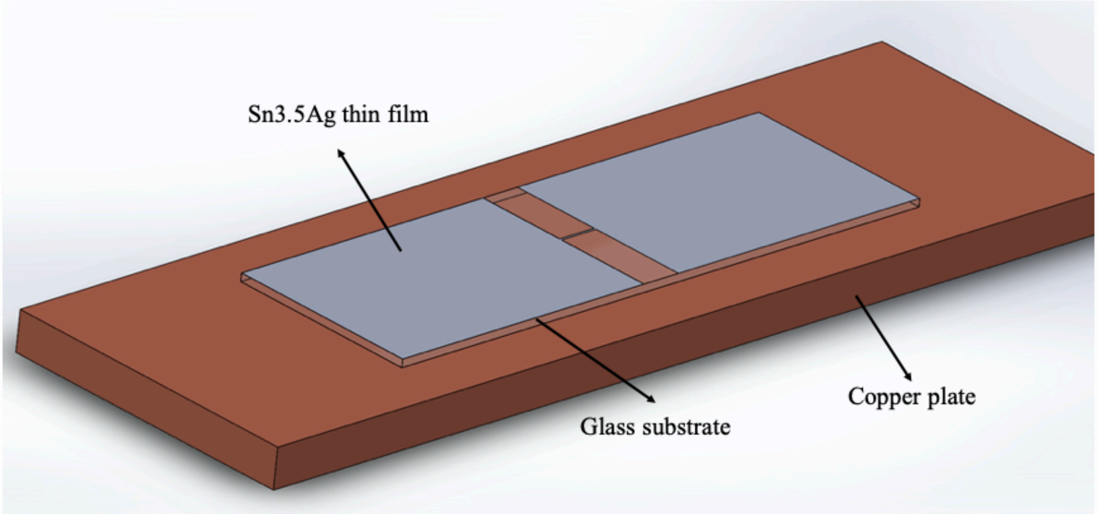

Figure S4. Model of specimen for simulation

Table S1: Parameters for the mathematic calculation

Given Parameters	values
Resistivity (ρ)	3.35×10^{-7} Ωm
Strip dimension	l:5 mm/w:300 um/h:300 nm
Voltage	1.3 V
Thermal conductivity (k)	630 (W/m^{-K})
Electrical conductivity (σ)	9.16×10^{6} (S/m)
